# The relationship between air pollution and multimorbidity: Can two birds be killed with the same stone?

**DOI:** 10.1007/s10654-022-00955-5

**Published:** 2023-01-16

**Authors:** Jorge Arias de la Torre, Amy Ronaldson, Jordi Alonso, Alex Dregan, Ian Mudway, Jose M. Valderas, Paolo Vineis, Ioannis Bakolis

**Affiliations:** 1 Health Service and Population Research Department, Institute of Psychiatry, Psychology and Neuroscience (IoPPN), Centre for Implementation Science, King’s College London, 16 De Crespigny Park, Camberwell, London, SE5 8AB UK; 2 CIBER Epidemiology and Public Health (CIBERESP), Madrid, Spain; 3 Institute of Biomedicine (IBIOMED), University of Leon, Leon, Spain; 4 Health Services Research Group, Hospital del Mar Medical Research Institute (IMIM), Barcelona, Spain; 5 Department of Medical and Life Sciences, Universitat Pompeu Fabra, Barcelona, Spain; 6 MRC Centre for Environment and Health, Environmental Research Group, Imperial College London, London, UK; 7 NIHR-HPRU Environmental Exposures and Health, School of Public Health, Imperial College London, London, UK; 8 Centre for Research in Health Systems Performance, National University Health System, Singapore, Singapore; 9 Psychological Medicine Department. Institute of Psychiatry, Psychology and Neuroscience (IoPPN), King’s College London, London, UK; 10 Department of Biostatistics and Health Informatics, Institute of Psychiatry, Psychology and Neuroscience, King’s College London, London, UK

**Keywords:** Air pollution, Multimorbidity, Public health, Health policy

## Abstract

Air pollution and multimorbidity are two of the most important challenges for Public Health worldwide. Although there is a large body of evidence linking air pollution with the development of different single chronic conditions, the evidence about the relationship between air pollution and multimorbidity (the co-occurrence of multiple long-term conditions) is sparse. To obtain evidence about this relationship could be challenging and different aspects should be considered, such as its multifaceted and complex nature, the specific pollutants and their potential influence on health, their levels of exposure over time, or the data that could be used for its study. This evidence could be instrumental to inform the development of new recommendations and measures to reduce harmful levels of air pollutants, as means to prevent the development of multimorbidity and reduce its burden.

## Introduction

According to the World Health Organization (WHO) and the Organisation for Economic Co-operation and Development (OECD) [1, 2], the joint effects of indoor and ambient air pollution air pollution are the main environmental cause of premature death, contributing between 4 and 9 million premature deaths per year [3], and with an associated economic cost of 1.575 trillion US dollars [2]. In addition, the exposure to air pollution in form of particulate matter (PM) of different sizes (e.g., with an average diameter of 10 µm or less—PM_10_, or with an average diameter of 2.5 µm or less PM_2.5_), or in form of gases (e.g., nitrogen dioxide—NO_2_, or Sulphur dioxide—SO_2_), has been directly related to a substantial decrease in the quality of life and to a substantial increase in the burden of multiple acute and chronic diseases [1, 4]. Following the recent WHO revision of the annual PM_2.5_ guideline value to 5 µg (one-millionth of a gram) per cubic meter air [5], it is estimated that over 99% of the world population is exposed to unhealthy levels of pollutants, further reinforcing air pollution as one of the main public health priorities worldwide.

Although different studies point out the relationship between the exposure to air pollution and the development of different single chronic conditions [6–13], the evidence about the relationship between air pollution and multimorbidity (the co-existence of two or more long-term health conditions) is very limited [14–17]. However, the nature of this relationship is complex and different peculiarities must be considered cautiously for its study, such as the potential mechanisms and pathways underlying it, the possible differential effects over time of the exposure to different pollutants and different levels of them, and their influence on the development of different multimorbidity clusters.

## Impact of air pollution on health conditions

Considering the large body of evidence linking air pollution with single conditions (e.g., cardiorespiratory, or neurological disorders) [6], it is reasonable to expect that the exposure to unhealthy levels of air pollution could also be related to their accumulation, leading to multimorbidity [18]. While there is considerable heterogeneity in the way multimorbidity has been defined in research [18, 19], all the definitions agree that multimorbidity is considered to be the co-occurrence of two or more long-term communicable and/or non-communicable diseases (or at least health problems) in an individual [14, 18]. Additionally, although multimorbidity has been linked systematically to different factors, such as biological (e.g., ageing and inflammation) [20], lifestyle (e.g., physical inactivity) [21], and psychosocial determinants (e.g., socioeconomic status) [22], there is sparse evidence for the potential role of air pollution in its development. Furthermore, air pollution could play a relevant role on the development and evolution of syndemics related to multimorbidity i.e., on the potential synergistic effect of the multimorbidity epidemic with other epidemics such as COVID-19. Therefore, the contribution of air pollution to the accumulation of different health conditions could translate into significant additional health, societal and economic burdens, currently not represented in the WHO and OECD assessments [1, 2].

The impact of air pollution on health conditions is likely to vary over time. While the short-term exposure to air pollution has been associated with the development of mainly, albeit not exclusively acute pathologies [23], the larger burden of health effects is attributable to longer-term, potentially lifetime exposures [24]. Exposure to pollutants could both increase the incidence and the severity of multiple long-term conditions (e.g., through the development of chronic inflammatory profiles [17], aberrant tissue remodelling, accelerated ageing and senescence) [25], making it necessary to carry out studies with long follow-up periods to determine the nature of this relationship over time. Additionally, the effects of air pollution on health could vary over the life course, necessitating the study and identification of both age groups in which the exposure could have more detrimental effects on health [26], and of sensitive periods in which the exposure to pollutants might have a greater adverse impact on health, such as during early immune and organ development [27].

## Dealing with complexity

It should be noted that the biological pathways underlying the relationship between the exposure to toxic air pollutants and the development of different types of multimorbidity needs further research [21, 28]. Since specific air pollutants are differentially related to the development of chronic health conditions [6], a wide range of air pollutants (such as NO_2_, SO_2_ and PM) and their pathways for the development of health problems must be considered over time. As highlighted by previous studies [29, 30], exposure to air pollutants in certain combinations could have different effects on the development of co-existing disorders and it remains unclear how they could impact multimorbidity via potential pathways, such as metabolic and neuroendocrine alterations, or systemic inflammation and oxidative stress [6, 25]. Furthermore, air pollutants are often co-occurring and collinear with each other and other socio-economic and environmental exposures, making it difficult to disentangle specific effects because they are intrinsically intertwined in a complex, dynamic network. To address the challenge of causality and collinearity, the exposome paradigm provides a framework that may advance the study of environment with the integration of omic markers in multimorbidity related epidemiological studies [31].

Due to the multifaceted and complex nature of the relationship between air pollution and multimorbidity, obtaining data to study it could be challenging. A suitable and promising option to study this relationship over time is the use of real-world data with existing or novel linkages with air pollution metrics [32]. Different types of real-world data from all over the world could be used to study this relationship, such as data population-based birth cohort studies, such as the National Survey of Health and Development (NSHD) in the UK [33], and the Danish National Birth Cohort (DNBC) in Denmark [34], or data from large-scale biomedical databases containing in-depth genetic and health information such as the UK Biobank in the UK [35], the “All of Us” Research Program in the US [36], or the Biobank for health research (Lifelines) in the Netherlands [37]. Furthermore, another promising option to be used for the study of the impact of air pollution on multimorbidity, are clinical data from electronic health records, such as the Clinical Practice Research Datalink (CPRD) [38].

Despite the opportunity offered by real-world data, it is worth mentioning that these data also have limitations [32]. In some cases, these data include a limited number of records or participants, and/or do not include variables potentially relevant to account for the complex relationship between air pollution and multimorbidity, as for example health records with a clinical focus in which could be limited the availability of non-clinical variables (e.g., sociodemographic, or environmental factors). Additionally, collecting relevant data about multi-morbidity and air pollution could be challenging in surveys or cohort studies. To account for them, these types of studies need to include data about a wide spectrum of physical and mental disorders, and about the exposure to air pollution over time (ideally on a continuous basis), or at least, proxy measures from which could be possible determine this exposure, such as the postcode of participants and possible changes on it [39]. To overcome these limitations and challenges, one possible solution is the use of pooled and/or linked real-world data from different sources. Different examples of real-world data pooling and linkage could be the European Study of Cohorts for Air Pollution Effects project (ESCAPE) that uses pooled data from more than 30 cohort studies with different variables of interest about the exposure to air pollution (determined using the postcode of cohort members), and about long-term conditions[39, 40], or a recent study aimed to study the influence of the exposure to air pollution on the development of different patterns of chronic diseases, that uses linked data from the UK Biobank and the Hospital Episodes Statistics database [16]. Therefore, data pooling and linkage could be a useful resource to enhance the richness, robustness and power of the data collected in individual studies to detect relevant relationships between air pollution and multimorbidity over the life course.

## Implications and conclusions

Mechanisms and challenges aside, if air pollution exposure affects multimorbidity risk, it presents an unusual opportunity for multimorbidity prevention: the possibility of integrating environmental policy and regulation into patient and healthcare policies aimed at addressing the multimorbidity epidemic [9, 10, 41–44]. This environmental perspective and population-wide approach could complement the individual perspective used in clinical settings [45], such as for the management of chronic diseases Furthermore, obtain evidence about the influence of air pollution on the development of multimorbidity, could be also helpful to determine the risk attributable to the exposure to different pollutants, and to identify high risk population groups. This evidence could be instrumental to inform the development of new environmental recommendations and preventive measures to reduce harmful levels of air pollutants, and to improve the outcomes of prevention strategies currently in use. Hence, obtain this evidence could suppose a step forward for cutting down substantial costs for healthcare systems, and for the prevention of the development of multiple co-occurring conditions [28].

Tackling multimorbidity represents one of the greatest challenges for health systems worldwide, and a deeper and more comprehensive understanding of its determinants is urgently needed. Therefore, given the lack of evidence, the study of the long-term influence of air pollution on the accumulation of chronic diseases would be key to inform preventive measures and, ultimately, reduce the burden of multimorbidity.
